# Sulforaphane Epigenetically Regulates Innate Immune Responses of Porcine Monocyte-Derived Dendritic Cells Induced with Lipopolysaccharide

**DOI:** 10.1371/journal.pone.0121574

**Published:** 2015-03-20

**Authors:** Xueqi Qu, Maren Pröll, Christiane Neuhoff, Rui Zhang, Mehmet Ulas Cinar, Md. Munir Hossain, Dawit Tesfaye, Christine Große-Brinkhaus, Dessie Salilew-Wondim, Ernst Tholen, Christian Looft, Michael Hölker, Karl Schellander, Muhammad Jasim Uddin

**Affiliations:** 1 Institute of Animal Science, University of Bonn, Endenicher Allee 15, 53115 Bonn, Germany; 2 Department of Animal Science, Faculty of Agriculture, Erciyes University, 38039 Kayseri, Turkey; 3 Department of Animal Breeding and Genetics, Faculty of Animal Husbandry, Bangladesh Agricultural University, Mymensingh-2202, Bangladesh; National Institutes of Health, UNITED STATES

## Abstract

Histone acetylation, regulated by histone deacetylases (HDACs) is a key epigenetic mechanism controlling gene expressions. Although dendritic cells (DCs) are playing pivotal roles in host immune responses, the effect of epigenetic modulation of DCs immune responses remains unknown. Sulforaphane (SFN) as a HDAC inhibitor has anti-inflammatory properties, which is used to investigate the epigenetic regulation of *LPS*-induced immune gene and HDAC family gene expressions in porcine monocyte-derived dendritic cells (moDCs). SFN was found to inhibit the *lipopolysaccharide LPS* induced HDAC6, HDAC10 and DNA methyltransferase (DNMT3a) gene expression, whereas up-regulated the expression of DNMT1 gene. Additionally, SFN was observed to inhibit the global HDAC activity, and suppressed moDCs differentiation from immature to mature DCs through down-regulating the CD40, CD80 and CD86 expression and led further to enhanced phagocytosis of moDCs. The SFN pre-treated of moDCs directly altered the *LPS*-induced TLR4 and MD2 gene expression and dynamically regulated the TLR4-induced activity of transcription factor NF-κB and TBP. SFN showed a protective role in *LPS* induced cell apoptosis through suppressing the IRF6 and TGF-ß1 production. SFN impaired the pro-inflammatory cytokine TNF-α and IL-1ß secretion into the cell culture supernatants that were induced in moDCs by *LPS* stimulation, whereas SFN increased the cellular-resident TNF-α accumulation. This study demonstrates that through the epigenetic mechanism the HDAC inhibitor SFN could modulate the *LPS* induced innate immune responses of porcine moDCs.

## Introduction

Apart from pork production, pig has been used as a major mammalian model in several fields of medical research because of the anatomy, physiology, metabolism, organ development and disease progression similarities to other mammalian species [1]. Importantly, pigs are the reservoirs of many zoonotic diseases make them important in the field of immunology. Therefore, deciphering of the porcine immune response is very important. Moreover, availability of numerous cell lines represent a broad range of tissues, further facilitates testing of gene expression and drug susceptibility of host immune system. Therefore, study of the porcine immune response could help to understand the immunological responses of the related mammalian species. Dendritic cells (DCs) play major roles at multiple layers of immune responses. DCs are professional antigen-presenting cells and primary phagocytic cells of innate immune system that induce both the innate and adaptive immune responses upon the detection of pathogens as well as maintain the immune tolerances. The porcine DC has been frequently used as an experimental model for studying the disease progression and pathogenesis after a highly contagious viral or bacterial infection in either human or swine viral infection physiology [2–6]. Such kind of external environmental stimuli can modify the epigenetic profile. This epigenetic modification may vary according to the cell types. We postulated that these epigenetic modifications may cause alterations of gene expression in porcine DCs in case of immune responses. The engagement of toll-like receptors (TLRs) by conserved microbial structures to activate the DCs is essential for initiation of innate immune response. *Lipopolysaccharide (LPS)*, the ligand of TLR4 but not other TLRs, as the most abundant component of Gram-negative bacterial cell wall has been extensively used in studying immune responses of mammalian cells. Because, *LPS* is the TLR4 agonist that activates nuclear factor-kappa B (NF-κB) and induces high-level of proinflammatory cytokines and chemokines expression after recognition by TLRs [7–9]. NF-κB plays an essential role in the regulation of transcription of genes related to rapid responses to stress and pathogens, as well as in the development and differentiation of immune cells (such as DCs and monocytes) [10]. Epigenetic modulation controls multi-layered interplay of NF-κB signalling pathway in achieving appropriate gene expression and transcriptional activity [11]. Previously, sulforaphane (SFN) has been found to regulate expressions of immune related gene [12–15].

SFN, a natural 1-isothiocyanato-4-(methylsufinyl)-butane compound present in cruciferous vegetables, exhibits anticancer and antimicrobial properties in experimental model [16,17], but the effects of SFN on cell growth, survival, and differentiation in primary cells are poorly understood. SFN is drawing great attention because of its ability to simultaneously modulate multiple cellular targets involved in cellular protection [18] and being suggested to be used in treatment of bacterial infection [17]. Additionally, previous studies reported that SFN had diminished HDAC activity, and both global and localized histone acetylation was increased [19,20]. HDAC enzymes remove acetyl groups from lysine residues within histones, which is important in the regulation of gene expression. The HDAC family has 11 (HDAC1-11) members which are catalogued in four classes: HDAC1-HDAC10 belong to class 1 and class 2 (as classic HDACs); a group of nicotinamide adenine dinucleotide (NDA+)-dependent proteins belongs to class 3 (called non classical HDACs), and the sole number of HDAC11 belongs to class 4. HDAC has been reported to affect the pro-inflammatory cytokines production in a range of disease models in mice, including septic shock [21,22]. Treatment of cells with SFN, as a HDAC inhibitor is regularly being used to investigate the role of histone modifications in the regulation of gene expressions [23]. Although, epigenetic modifiers, such as HDAC inhibitors have considerable potential as anti-inflammatory and immunosuppressive agents, their effect on porcine DCs has not yet been deciphered. The epigenetic effects of SFN on porcine DCs could extend our knowledge to understand the mechanism of epigenetic regulation in human antigen-presenting cells.

The monocyte-derived dendritic cells (moDCs) have been established *in vitro* as an ideal culture model to examine the DCs function [24]. The epigenetic effect of SFN has been studied in various tissues and cells in mice and humans [20,25]. Effects of HDAC inhibitors in *LPS*-induced innate immune response have never been reported in porcine DCs. Therefore, this study aimed to investigate the effect of the HDAC inhibitor, SFN on the *LPS* induced inflammatory response in porcine moDCs. For this purpose, the effects of *LPS* stimulation on expression of genes encoding HDACs and DNA methyltransferases and acetylation levels were analysed. Furthermore, the modulations of SFN on *LPS*-induced inflammatory response and TLR4 activation are also examined.

## Materials and Methods

### Animals

Three 35 days old Pietrain female piglets were housed at the Teaching and Research Station of Frankenforst, University of Bonn, Germany. All the piglets were clinically healthy and no respiratory disease was found according to the clinical history and physical examinations. The feeding, housing and husbandry practices of the animals followed the ‘Guideline for performance testing of pigs on station for production and carcass traits (ZDS, 2003)’. This experiment was approved and followed the guidenline of ‘Richtlinie Fuer die Stationspruefung auf Mastleistung, Schlachtkoerperwert und Fleischbeschaffenheit Beim Schwein. Zentralverband der Deutschen Schweineproduktion eV, Ausschussfuer Leistungspruefung und Zuchtwertschaetzung, Bonn’ (Central Board of the German Pig Producers ev. Committee for Performance Testing, Animal Breeding Value Estimation, Bonn, Germany) [26]. Moreover, this study was carried out in strict accordance with the recommendations in the Guide for Animal Welfare committee of the University of Bonn with proposition number 84-02.05.20.12.075.

### Generation of moDC from adherent monocytes of PBMCs

Porcine blood samples were collected from the vena cava cranialis in sterilized tubes with ethylenediaminetetraacetic acid (EDTA) which were used to isolated peripheral blood mononuclear cells (PBMCs). The PBMCs were isolated by Ficoll-histopaque (cat. 10771, Sigma, Germany) using density gradient centrifugation as described previously [27,28]. PBMCs were washed two times in cold Dulbecco's Phosphate Buffered Saline (DPBS) (cat. 14190-094; Invitrogen, Germany) and re-suspended in Dulbecco’s modified Eagle medium (DMEM) (cat. 41966-029; Invitrogen, Germany) supplemented with 2% fetal bovine serum (FBS) (cat. 10270; Invitrogen, Germany), 500 IU/ml Penicillin-Streptomycin (cat. 15140; Invitrogen, Germany) and 0.5% fungizone (cat. 15290-026; Invitrogen, Germany). PBMCs (5 × 10^6^ cell/ml) were cultured in 6-well plate (2 ml/well) for 4 h. The moDCs were generated from the adherent monocytes following the procedure described previously [29–31]. Briefly, PBMCs were incubated for 4 h, non-adherent cells were discarded by vacuum aspiration and the adherent monocytes were washed two times using pre-warmed (37°C) DPBS in order to remove the non-adherent cells. The cleaned monocytes were cultured in RPMI-1640 medium (cat. 21875; Invitrogen, Germany) supplemented with 10% FBS, 1000UI/ml Penicillin-Streptomycin, 1% fungizone, 20 ng/ml recombinant porcine (rp) granulocyte-macrophage colony-stimulating-factor (GM-CSF) (cat. 711-PG-010; R&D System, UK) and 20 ng/ml recombinant porcine (rp) interleukin-4 (IL-4) (cat. 654-P4-025; R&D System, UK) for 7 days at 37°C with 5% CO_2_. Half of the medium was replaced every 3rd day with the fresh medium supplemented the rp GM-CSF (20 ng/ml) and rp IL-4 (20 ng/ml) concentration. After 7 days of incubation the adherent moDCs were counted and re-cultured in a new plate for the subsequent assays.

### Stimulation of moDCs

moDCs were seeded separately at 2 × 10^6^ cells/well into 6-well tissue culture plates and incubated over night at 37°C in 5% CO_2_ incubator. Afterwards, moDCs were treated with or without SFN (cat. LKT-8044, LKT Laboratories, Inc., Germany) at the concentrations of 5 μM, 10 μM, 15 μM, 20 μM and 50 μM for 24 h for either cells viability or HDAC activity assay. For the gene expression study, inflammatory cytokines and other proteins measurement, cells were pre-incubated with or without 10 μM SFN for 24 h prior to the stimulation with *LPS* (1μg/ml) (cat. tlrl-3eblps, Invitrogen, France) for additional 24 h or indicated time (such as 0, 1, 3, 6, 12, 24 h). Cells were harvested after 24 h or indicated time of *LPS* stimulation. Total RNA was isolated from these cells for gene expression analysis and the supernatants were used to measure the cytokine levels. Similarly, for the expression of co-stimulatory molecules (CD40, CD80, and CD86) of cells, moDCs were pre-incubated with or without SFN before stimulating with 1 μg/ml *LPS* for 24 h. In addition, the effects of SFN (10 μM) on the phagocytic activity of moDCs were determined following stimulation with different concentration of *LPS* (0.5 μg/ml, 1.0 μg/ml, and 2.0 μg/ml) for 4 h.

### Cell viability assay

Cell viability was investigated using the WST-1 cell proliferation kit (cat. 10008883, Cayman Chemical) following to the manufacturer’s instructions as described previously [24]. For the dose dependent SFN effect on cell viability assay, moDCs were cultured with SFN at the concentration of 5 μM, 10 μM, 15 μM, 20 μM and 50 μM for 24 h. In order to study the effect of SFN on *LPS* induced moDCs death, moDCs pre-incubated with or without SFN (10 μM) for 24 h, were stimulated with or without *LPS* at the concentration of 1.0 μg/ml for indicated time 1, 3, 6, 12 and 24 h. Then, 10 μl of reconstituted WST-1 mixture was added to each well. After 2 h of incubation in a CO_2_ incubator at 37°C, the absorbance of the samples was measured using a microplate reader (Thermo max; Germany) at a wavelength of 450 nm. The cell viability was calculated (%) following the manufacturer’s formula.

### DCs maturation measurement using flow cytometry

For the flow cytometry (FACs) analysis, moDCs were pre-cultured with SFN (10 μM) for 24 h before stimulation with *LPS* (1.0 μg/ml) for 24 h. The cells were harvested and incubated for 30 min with FACs staining buffer (DPBS supplement with 2% FBS, 10 mM NaN_3_ and 10 mM HEPES) and then washed with the same staining buffer. Cells were stained with a mouse anti-human CD40 FITC Ab (clone G28.5, NB100-77786, Novus Biologicals), a mouse anti-human CD80 PE Ab (clone 37711, FAB140P, R&D systems) and a mouse anti-human CD86 APC Ab (clone 37301, FAB141A, R&D systems) antibodies. All these antibodies were IgG1 isotype. Cells were stained for 30 min on ice in 100 μl of staining buffer solution in a light protected condition. The events were acquired on 10,000 cells using a FACscalibur Dual Laser Flow Cytometer (BD Biosciences; USA) and analysed by FlowLogic software (BD Biosciences; Germany).

### Gene expression analysis

mRNA expression of the genes of interest was quantified using qRT-PCR (quantitative real time PCR). Total RNA was isolated using miRNeasy Mini Kit (cat. 217004; Qiagen, Germany) and the RNA concentration was measured by Nanodrop 8000 (Thermo Scientific, Pittsburgh, PA, USA). Complementary DNA (cDNA) was synthesized using miScript II RT kit (cat. 218161; Qiagen, Düsseldorf, Germany) and the cDNA was stored at −20°C for further use. qRT-PCR was performed in an ABI prism7000 (Applied Biosystems, Darmstadt, Germany) qRT-PCR system. The transcript of target genes presented in each sample was determined using Maxima SYBR Green/ROX Mix (cat. 218073; Qiagen, Düsseldorf, Germany). The primers (Table 1) were designed using the online Primer3 (version 0.4.0) [32]. The qRT-PCR was conducted with the following program: 95°C for 3 min, 40 cycles at 95°C for 15s, 60°C for 1 min and 95°C for 1 min in the StepOne Plus qPCR system (Applied Biosystem, Germany). Melting curve analysis was performed to detect the specificity of the PCR reaction. Each experiment was performed in triplicates and each sample was quantified in triplicate (technical replication) using qRT-PCR. Gene-specific expression was measured as relative to the expression of the house keeping gene hypoxanthine phosphoribosyltransferase 1 (HPRT1) (Table 1). The delta Ct (ΔCt) values were calculated as the difference between target gene and reference gene HPRT1. The average expression values were considered for further analysis.

**Table 1:** List of primer sequences used in this study.

*Gene*	Primer set	Annealing temperature (°C)	Amplicon size (bp)	GenBank accession number
*HDAC1*	F: GGAAATCTATCGCCCTCACAR: AAACACCGGACAGTCCTCAC	60	157	XM_003356305.2
*HDAC2*	F: AACCTGCTGCTTGGAGAAAAR: ACCATCAGGATGCAAAGCTC	60	201	XM_001925318.3
*HDAC3*	F: CAACCAGGTGGTGGACTTCTR: GCAGAGGGATGTTGAAGCTC	60	152	NM_001243827.1
*HDAC4*	F: GGTCCTCGCCTACCTTATCCR: GACGCCTGGTAGTTCCTCAG	60	189	XM_003359701.2
*HDAC5*	F: AGATGCACTCCTCCAGTGCTR: GGATGATGGCAAATCCATTC	60	102	XR_135351.1
*HDAC6*	F: ATGGACGGGTATTGCATGTTR: GCGGTGGATGGAGAAATAGA	60	168	XM_003360315.2
*HDAC7*	F: CGTCCCCTACAGAACTCTCGR: TCAGGTTGGGCTCAGAGACT	60	146	XM_003355640.2
*HDAC8*	F: GGTGACGTGTCTGATGTTGGR: AGCTCCCAGCTGTAAGACCA	60	165	XM_003360365.2 165
*HDAC9*	F: AACTGAAGCAACCAGGCAGTR: CCCAACTTGTCCCAGTGAGT	60	149	XM_003122063.2
*HDAC10*	F: TCCATCCGAGTACCTTCCACR: GGCTGCTATGGCCACACTAT	60	179	XM_003362070.1
*HDAC11*	F: GACAAGCGCGTGTACATCATR: AGGTTCCTCTCCACCTTCGT	60	143	XM_003483230.1
*DNMT1*	F: GCGGGACCTACCAAACATR: TTCCACGCAGGAGCAGAC	60	133	DQ060156
*DNMT3a*	F: CTGAGAAGCCCAAGGTCAAGR: CAGCAGATGGTGCAGTAGGA	60	238	NM_001097437
*CD40*	F: TGAGAGCCCTGGTGGTTATCR: CTCTCTTTGCCATCCTCCTG	60	235	NM_214194.1
*CD80*	F: TCAGACACCCAGGTACACCAR: GACACATGGCTTCTGCTTGA	60	189	NM_214087.1
*CD86*	F: TTTGGCAGGACCAGGATAACR: GCCCTTGTCCTTGATTTGAA	60	152	NM_214222.1
*TLR4*	F: ATCATCCAGGAAGGTTTCCACR: TGTCCTCCCACTCCAGGTAG	58	235	NM_001097444.1
*MD2*	F: TGCAATTCCTCTGATGCAAGR: CCACCATATTCTCGGCAAAT	60	226	NM_001104956.1
*NF-κB1*	F: TGGGAAAGTCACAGAAACCAR: CCAGCAGCATCTTCACATCT	60	187	NM_001048232.1
*TBP*	F: GATGGACGTTCGGTTTAGGR: AGCAGCACAGTACGAGCAA	60	124	DQ845178.1
*IFN-γ*	F:AGCTCCCAGAAACTGAACGAR:AGGGTTCAAAGCATGAATGG	60	225	NM_213948.1
*TNF-α*	F: CCACCAACGTTTTCCTCACTR: CCAAAATAGACCTGCCCAGA	60	247	NM_214022.1
*IL-1ß*	F: GTACATGGTTGCTGCCTGAAR: CTAGTGTGCCATGGTTTCCA	59	137	NM_001005149.1
*IL-8*	F:TAGGACCAGAGCCAGGAAGAR:CAGTGGGGTCCACTCTCAAT	60	174	NM_213997.1
*HPRT1*	F: AACCTTGCTTTCCTTGGTCAR: TCAAGGGCATAGCCTACCAC	60	150	NM_001032376.2

F: Forward primer; R: Reverse primer; bp: base pair.

### Nuclear extraction and in vitro HDAC activity assay

moDCs were cultured with SFN (0 μM, 5 μM, 10 μM, 15 μM, 20 μM and 50 μM) for 24 h. After that, cells were harvested using trypsin-EDTA (cat: 25200-072; Invitrogen, Germany). The nuclear extracts were obtained from cultured cells according to the manufacturer’s instruction. Briefly, 500 μl of ice-cold hypotonic lysis buffer (10 mM HEPES, 1.5 mM MgCl_2_, 10 mM KCl, 0.5 mM DTT, 0.05% NP40 [or 0.05% Igepal or Tergitol] pH 7.9) containing 1% proteinase inhibitor (cat: P8340-1ML; Sigma, Germany) was added to approximate 4 × 10^6^ trypsinized cells. Cells were lysed using a mechanical homogenizer on ice for 20 min and centrifuged at 3000 rpm for 10 min at 4°C. The cell pellets were re-suspended in 374 μl of buffer (5 mM HEPES, 1.5 mM MgCl2, 0.2 mM EDTA, 0.5 mM DTT, 26% glycerol [v/v], pH 7.9) containing 26 μl of 4.6 M NaCl and homogenized with 20 abundant strokes on ice. The cell pellets were lysed for 30 min on ice and then centrifuged at 14,000 g for 20 min at 4°C. The supernatants containing the nuclear extract were removed and stored at −80°C for subsequent analysis. Protein content was determined using Bradford assay.

*In vitro* HDAC activity was determined using the Color-de-Lys HDAC colorimetric activity assay kit (BML-AK501-0001, Enzo Life Sciences) according to the manufacturer’s instructions. Briefly, approximately 5 μg of nuclear fraction from each treatment samples was incubated with the HDAC assay buffer and the HDAC colorimetric substrate at 37°C for 30 min. Then the lysine developer was added and the samples were incubated at 37°C for another 30 min. At the end of the incubation period, readings were taken at 405 nm using an ELISA plate reader (ThermoMax, Germany).

### Phagocytic activity assay

Phagocytosis of the cells was investigated using Vybrant Phagocytosis Assay kit (cat. V-6694, Molecular Probes, Germany). The principle of this analysis is based on the intracellular florescence emitted by the engulfed particles, as well as the effective fluorescence quenching of the extracellular probe by trypan blue [33]. The phagocytosis assay protocol follows the instruction of using five negative controls, five positive controls and four experimental samples. Briefly, cells were cultured in a 6-well cell culture plate for 48 h with RPMI-1640. Cells were scrapped, washed twice with DPBS and cell density was adjusted to 1 × 10^6^ cells/ml. moDCs were pre-treated with SFN (10 μM) for 24 h before stimulating with *LPS*. For the phagocytosis assay, 50 μl of the *LPS* (at final concentration of 0.5 μg/ml, 1 μg/ml, 2 μg/ml in RPMI-1640) were added to the experimental wells containing cells. The cells were incubated for 4 h at 37°C with 5% CO_2_ to allow the cells to adhere on the microplate surface. Afterwards, the RPMI-1640 solution was removed from the microplate wells. Then 100 μl of fluorescent BioParticles suspension was added to all the negative control, positive control and experimental wells. Two hours after incubation at 37°C in CO_2_ incubator, the BioParticles were aspirated from all of the microplate wells. Finally, 100 μl trypan blue was added to the wells and incubated for 1 min at room temperature, and then trypan blue was aspirated. The fluorescence emission was measured in a fluorescence microplate reader (Thermo Eectron, Waltham, MA, USA) using 480 nm excitation and 520 nm emissions. The net phagocytosis of the cells was calculated according to the response of the phagocytosis effector agent following the manufacturer’s instructions.

### Cytokines measurement by ELISA

ELISA was used to investigate the cytokines secretion differences of moDCs in different treatment groups. 1 × 10^6^ cells/ml was cultured in 6-well plates. moDCs were pre-incubated with SFN (10 μM) for 24 hour before stimulating with 1 μg/ml *LPS* for 24 h. Supernatants were collected for TNF-α (cat. PTA00; R&D Systems, UK) and IL-1ß (cat. PLB00B; R&D Systems, UK) measurements using ELISA kits following manufacturer’s instructions. The optical density (OD) values were measured by microplate reader (ThermoMax; Germany) setting to 450 nm of wave length and results were calculated according to manufacturer’s formula.

### Western blot

Cell lysates were electrophoresed through polyacrylamide gels and transferred onto nitrocellulose membranes. Membranes were incubated with polyclonal antibodies specific for NF-κB p65 (Cat. ab72555; abcam; UK), TGFß1 (cat. sc-146; Santa Cruz Biotechnology, Inc; Germany), IRF6 (cat. sc-98829; Santa Cruz Biotechnology, Inc; Germany), TNF-α (cat. LS-C43037; LSBio; Germany) and ß-actin (cat. sc-47778; Santa Cruz Biotechnology, Inc; Germany), and then revealed with secondary antibody. As a secondary antibody, horseradish peroxidase-conjugated goat anti-rabbit (cat. Sc-2004; Santa Cruz Biotechnology, Inc; Germany) was used for the primary antibody of NF-κB p65, IRF-6, and TNF-α; whereas, peroxidase-conjugated goat anti-mouse IgG (cat. Sc-2005; Santa Cruz Biotechnology, Inc; Germany) was used for ß-actin primary antibody. Mouse polyclonal anti-ß-actin antibody was used to correct minor differences in protein loading. Finally, the specific signals were detected by chemiluminescence using the SuperSignal West Pico Chemiluminescent Substrate (cat. 34077, Thermo Scientific, Germany). Images were acquired by Quantity One 1-D analysis software (Bio-Rad, Germany).

### Statistical analysis

The data were analysed by SAS software package ver. 9.2 (SAS institute, Cary, NC, USA). Pairwise comparisons were made between the treatment groups and control, using Student’s t test. In addition, to compare multiple treatments groups a variance analysis was followed by Tukey test. The data were expressed as means ± standard deviations (SD) and (*) *P* < 0.05, (**) *P* < 0.01, (***) *P* < 0.001 were set as statistically significant.

## Results

### LPS treatment differentially influences HDACs gene expression

**Fig 1:**
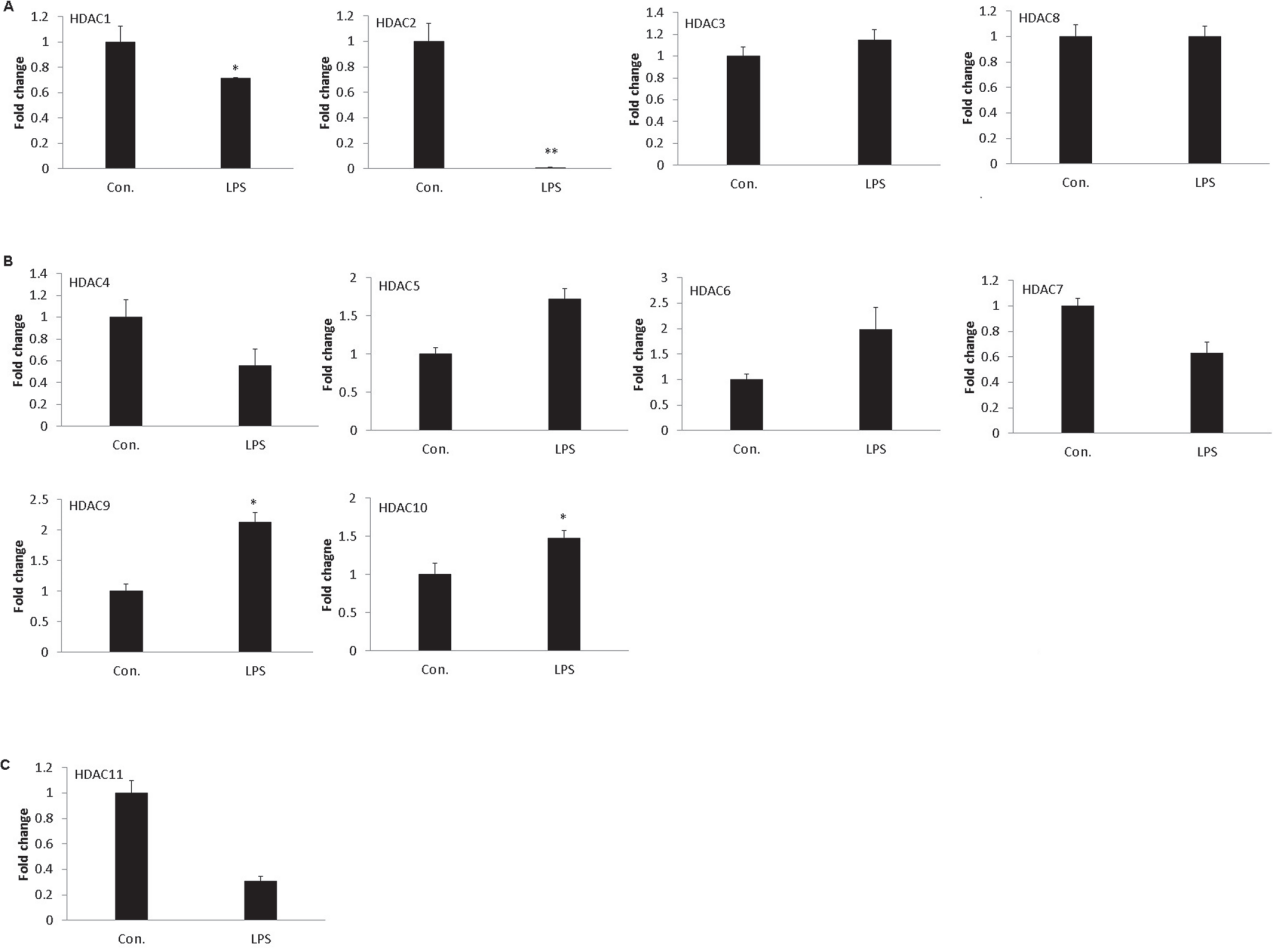
Expression of class I, class II, and class IV HDAC genes in porcine moDCs stimulated with LPS. The expression of HDAC family genes in moDCs were influenced with LPS (1μg/ml) stimulation for 24 h. moDCs were generated from adherent monocytes at day 7 *in vitro*, which were treated with or without LPS. The class I (A), class II (B), and class IV (C) of HDACs mRNA were quantified by qRT-PCR and normalized with the housekeeping gene HRPT1. The results were combined from three independent experiments and each experiment performed in triplicate. The data were represented as the mean ± standard deviations (SD) (* *P* < 0.05; ** *P* < 0.01).

The expression profiling of genes encoding the four classes of HDAC were quantified by the qRT-PCR. After 24 h *LPS* exposure, class 1 HDACs such as HDAC1 and HDAC2 mRNA were significantly down-regulated in moDCs (Fig. 1A). On the other hand, HDAC9 and HDAC10, belonging to the class 2 HDAC, were remarkably up-regulated after *LPS* stimulation for 24 h (Fig. 1B). In addition, the unique HDAC11 belonging to class 4 HDAC was numerically decreased, but did not show statistically significant differences (Fig. 1C). While *LPS* stimulation altered the expression of five HDAC genes, six HDAC gene expressions remained non-significant in moDCs in response to *LPS* stimulation (Fig. 1).

### Effect of LPS stimulation on DNA methyltransferase (DNMT) gene expressions in moDCs

**Fig 2:**
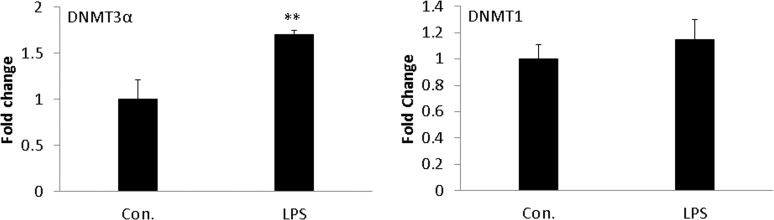
The effects of LPS on DNMT gene expression. Expression of genes that encode the enzymes responsible for methylating CpG sites of DNA were quantified by qRT-PCR including the maintenance methyltransferase DNMT1 and the *de novo* methyltransferase DNMT3a in moDCs in response to LPS exposure (24 h) compared with control. The results were combined from three independent experiments and each experiment was performed in triplicate. The data were represented as the mean ± standard deviations (SD) (* *P* < 0.05; ** *P* < 0.01).

Expression of genes encoding the enzymes responsible for methylating CpG sites in their DNA recognition elements was also analysed in *LPS*-induced porcine moDCs which included the maintenance methyltransferase DNMT1 and the de novo methyltransferases DNMT3a and DNMT3b. Expression of all three transcripts (DNMT1, DNMT3a and DNMT3b) were investigated in both untreated and *LPS* treated cells (Fig. 2). The expression of the DNMT1 was found to be unaffected by *LPS* treatment, in contrast, the *de novo* methyltransferase genes DNMT3a showed up-regulation in *LPS* stimulated moDCs. Notably, in this study, DNMT3b was undetectable in porcine moDCs.

### Effect of SFN on moDCs viability and LPS-induced cell death

**Fig 3:**
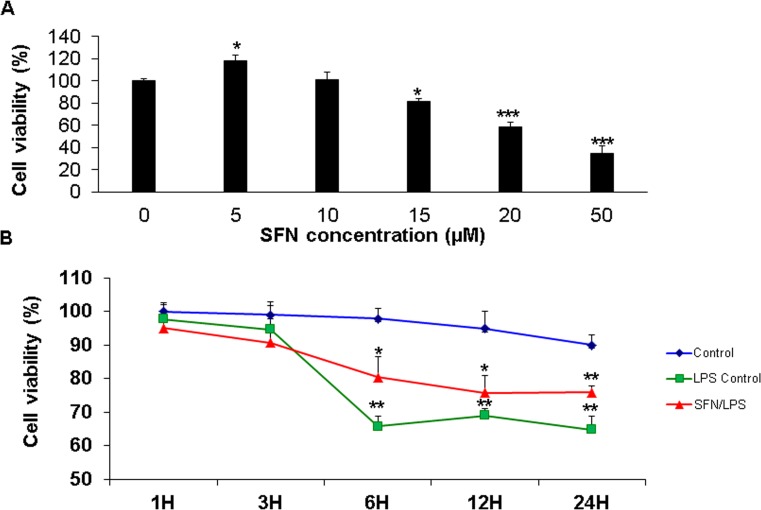
SFN pre-incubation inhibited LPS induce cell death in a time denpendent manner. moDCs at day 7 were used for cell viability assay by WST-1 kit. A SFN dose-dependent assay was used to confirm cell viability of moDCs after stimulating with different concentration of SFN (Control, 5 μM, 10 μM, 15 μM, 20 μM, and 50 μM) for 24h (A). For the effects of SFN on LPS induced cell death, moDCs were pre-incubated 24 h with or without SFN (10 μM) before exposed to LPS (1 μg/ml) for 1, 3, 6, 12, and 24 h (B). The results were combined from three independent experiments and each experiment was performed in triplicate. The data were represented as the mean ± standard deviations (SD) (* *P* < 0.05; **P<0,01; ***P<0,001).

In order to examine the effects of SFN treatment on moDCs viability, cell viability was measured using WST-1 cell proliferation kit as described in earlier section. The moDCs viability was decreased significantly after exposure to the higher dose of SFN (15 μM and 20 μM), while cell viability remained unaffected in response to the lower dose of SFN (5 μM and 10 μM) (Fig. 3A) evidencing a dose-dependent effect of SFN on moDCs viability. In addition, 1 μg/ml *LPS* dramatically induced cell death after 3 h *LPS* stimulation (Fig. 3B). Notably, the SFN (10 μM) pre-incubation significantly inhibited the *LPS*-induced cell death after at 3 h *LPS* stimulation (Fig. 3B).

### Regulation of genes encoding epigenetic enzymes by SFN

**Fig 4:**
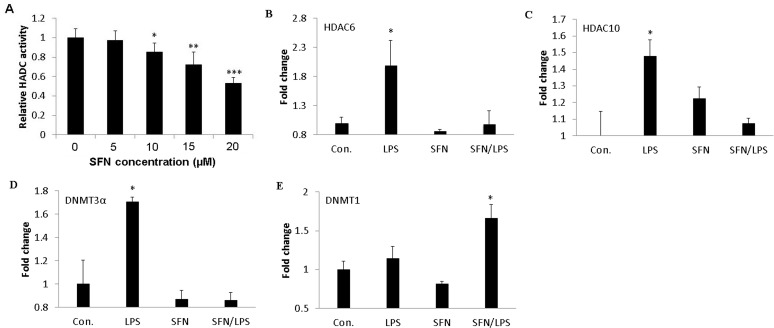
SFN inhibits HDAC activity and regulates genes which encode epigenetic enzymes. moDCs at day 7 in cell culture were used for this experiment. Relative HDAC activity assay was measured using the Color-de-Lys HDAC colorimetric activity assay kit. To confirm the global HDAC deacetylation of moDCs, cells were stimulated with different concentration of SFN (Control, 5 μM, 10 μM, 15 μM, and 20 μM) for 24 h (A). Equal amounts of isolated nuclear protein were subjected to HDAC activity analysis. The effects of SFN (10 μM) on gene expression of epigenetic encoding enzymes in porcine moDCs stimulated with LPS were examined. The HDAC6 (B), HDAC10 (C), DNMT3a (D) and DNMT1 (E) mRNA expression was quantified using qRT-PCR. The moDCs were pre-treated for 24 h with or without SFN before stimulating with LPS (1 μg/ml) for additional 24 h. The results (A, B, C, D, and E) were represented as the mean ± standard deviation (SD) of three independent experiments and each experiment was performed in duplicate (*p < 0.05; **p < 0.01; ***p < 0.001).

We investigated the HDAC activity in different concentration of SFN treatments. The results showed that SFN significantly inhibited HDAC activity in a dose-dependent manner (Fig. 4A). The effects of SFN on genes encoding epigenetic enzyme showed that SFN treatment caused a decrease in mRNA expression of several HDAC genes in *LPS* treated porcine moDCs (Fig. 4B and C). Our results show that SFN significantly inhibited both HDAC6 and HDAC10 mRNA expression that were induced by *LPS* in moDCs (Fig. 4B and C). Similarly, SFN treatment significantly enhanced the down-regulation of *de novo* methyltransferase DNMT3a that was induced by *LPS* treatment (Fig. 4D). The DNMT1 expression was significantly increased in SFN pre-treated moDCs that was induced by *LPS* treatment (Fig. 4E). However, this trend could not be observed in the case of other (data not shown) DNMT genes.

### Effect of SFN treatment on the maturation status and phagocytosis of moDCs

**Fig 5:**
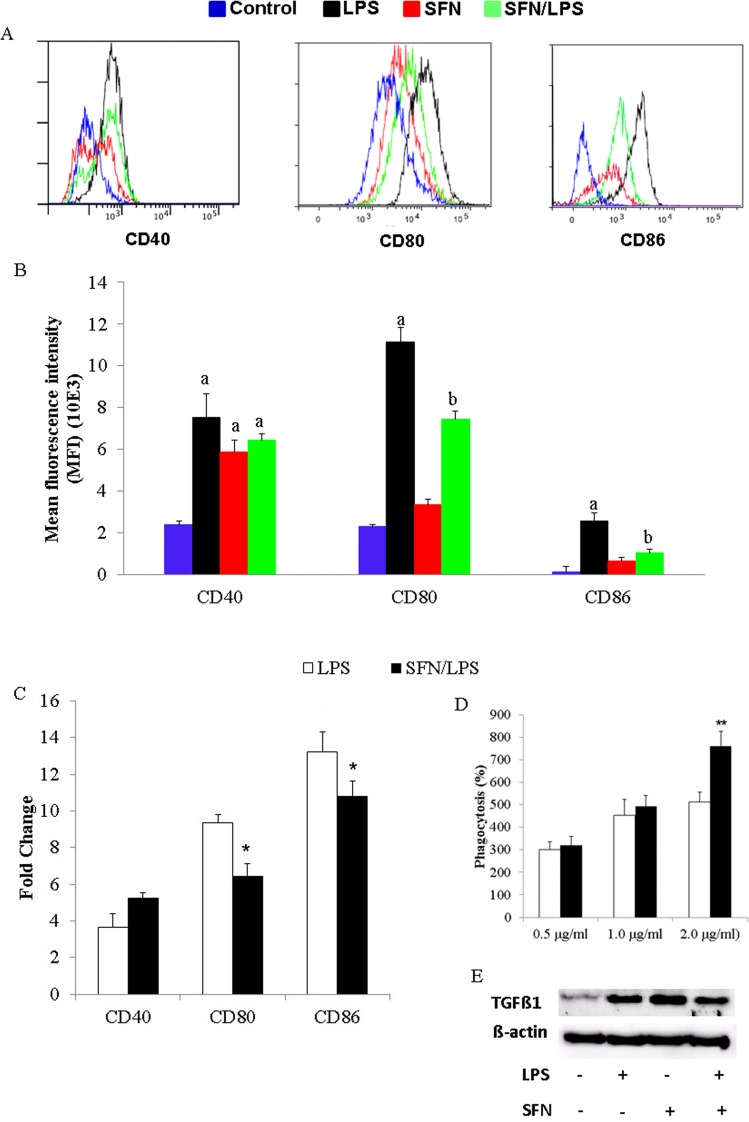
SFN inhibits LPS induced moDC maturation and enhances the phagocytic activity. moDCs at day 7 in culture were used for cell phagocytosis and cell differentiation status analysis. moDCs were pre-incubated for 1 h with or without SFN (10 μM) before stimulation for 24 h LPS (1.0 μg/ml) or to the indicated concentrations. CD40, CD80, and CD86 cellular surface markers expression were analyzed by flow cytometry (A). The flow cytometry results shown were from one experiment of two independent experiments. CD40, CD80 and CD86 mean fluorescence intensity (MFI) determined by flow cytometry (B). The flow cytometry results were combined from two independent experiments and each experiment was performed from triplications. Data are mean ± standard deviations (SD) (the letters a and b *P<*0.01). The phagocytic activity of moDCs was examined after stimulating with different concentration of LPS (0,5 μg/ml, 1,0 μg/ml, and 2,0 μg/ml) with or without 24 h pre-treatment with SFN (C). The mRNA expression of DCs surface markers CD40, CD80 and CD86 were quantified using qRT-PCR (D). The mRNA expression and phagocytosis results were combined from three independent experiments and each experiment was performed in four replications. The data represented as the mean ± standard deviations (SD) (* *P* < 0.05; ** *P* < 0.01; *** *P* < 0.001).

In order to determine the influences of SFN on *LPS*-induced moDC maturational status, we examined the co-stimulating molecules CD40, CD80/86 expression. The flow cytometry results showed that *LPS* dramatically induced the expression of CD40 and CD80/86 molecules on with or without SFN pre-incubated moDCs (Fig. 5A and 5B). Furthermore, the SFN pre-incubation significantly inhibited CD80/86 molecule expression on moDCs (Fig. 5A and 5B). Besides, we further performed expression of those molecules at mRNA level, CD40 and CD80/86 mRNA expression was quantified using qRT-PCR. The SFN significantly inhibited the *LPS*-induced co-stimulatory molecules CD80 and CD86 gene expression in moDCs (Fig. 5C). Additionally, the effects of SFN on the potential of phagocytic activity were also measured. The effects of SFN and *LPS* on phagocytosis showed a dose-dependent manner (Fig. 5D). The phagocytosis activity of moDCs was increased with an increase of both the SFN and *LPS* dose (Fig. 5D). SFN pre-treatment significantly increased the phagocytosis of moDCs in response to 2.0 μg/ml of *LPS* (Fig. 5D) treatment. Moreover, the western blotting result displayed that TGFß1 secretion was significantly increased in response to either SFN and/or stimulation group compared to control group (Fig. 5E). Moreover, the SFN pre-treatment suppressed TGFß1 production in response to *LPS* stimulation (Fig. 5E).

### SFN reversed LPS-induced up-regulation of TLR4 and MD2 gene expression in the early stage of LPS stimulation

**Fig 6:**
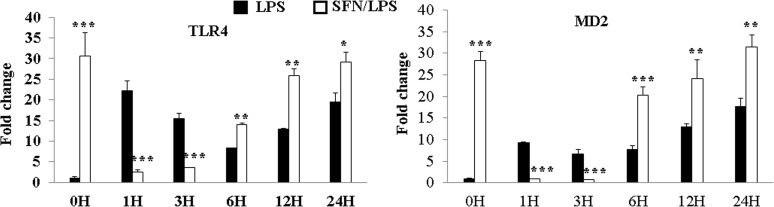
The effects of SFN on LPS induced up-regulation of TLR4 and MD2 gene expression. moDCs were pre-incubated for 1 h with or without SFN (10 μM) before stimulation for 0, 1, 3, 6, 12, 24 h with LPS (1.0 μg/ml). The TLR4 and MD2 mRNA expression were quantified by qRT-PCR. The results were combined from three independent experiments and each experiment was performed in four replications. The data represented as the mean ± standard deviations (SD) (* *P* < 0.05; ** *P* < 0.01; *** *P* < 0.001).

To clarify how SFN influences TLR4 activation in the time-dependent manner of *LPS* stimulation, moDCs were pre-incubated with SFN (10 μM) for 24 h and then stimulated with or without *LPS* for the indicated times. It was found that SFN significantly up-regulated TLR4 and MD2 mRNA expression following 24 h SFN incubation (Fig. 6). Interestingly, SFN significantly inhibited *LPS*-induced up-regulation of TLR4 and MD2 within the first 3 h of *LPS* stimulation (Fig. 6). Surprisingly, after 6 h *LPS* stimulation, SFN dramatically enhanced *LPS*-induced up-regulation of TLR4 and MD2 (Fig. 6).

### SFN reversed LPS-activated transcription factor expression in a time-dependent manner

**Fig 7:**
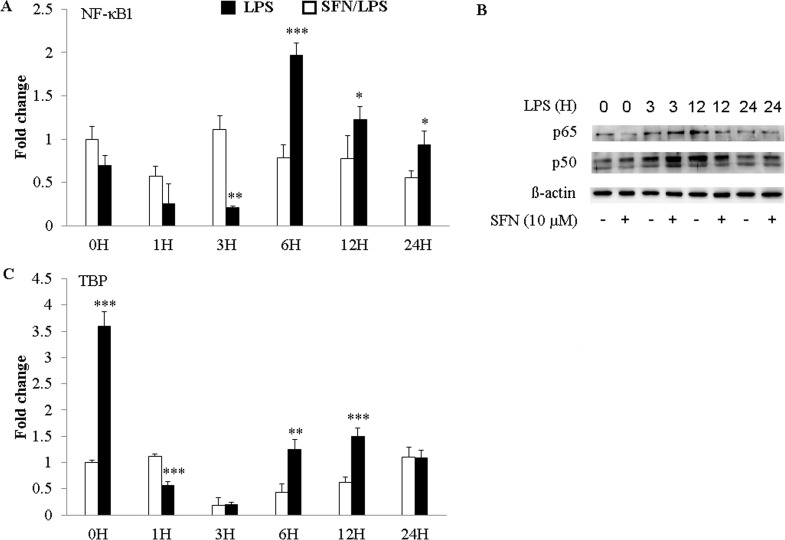
The effects of SFN on LPS induced NF-κB and TBP expression. moDCs were pre-incubated for 1 h with or without SFN (10 μM) before exposure to LPS (1.0 μg/ml) for 0, 1, 3, 6, 12, 24 h or to the indicated time. The transcription factor NF-κB and TBP mRNA expression were quantified by qRT-PCR (A and C). Data are mean ± standard deviations (SD) (* *P* < 0.05; ** *P* < 0.01; *** *P* < 0.001) of triplication samples from three independent experiments. NF-κB and TBP protein expression were examined by western blotting. The results were determined from one experiment representative of two experiments. The p50 and p65 protein of NF-κB family were analyzed by western blotting by the selected time points (0, 3, 12, and 24 h) (B). The western blotting result was from one experiment of three independent experiments.

In order to further confirm the time-dependent regulation of SFN in *LPS* induced immune response, we have examined the transcription factor NF-κB1 and TBP expression which are present in most mammalian immune cells such as in moDCs. It could be found that SFN significantly inhibited *LPS*-induced up-regulation of NF-κB1 mRNA at 3 h (Fig. 7A). Since the classic NF-κB typically presents as a p50-p65 heterodimer structure in the cytoplasm, we have detected the effects of SFN on *LPS*-activated p50 and p65 expression at protein level. The western blotting results showed that SFN visibly impaired p65 production, whereas SFN enhanced p50 secretion in moDCs (Fig. 7B). *LPS* up-regulated p65 was dramatically inhibited at 3 h, but was enhanced at 6 h by SFN pre-incubation in porcine moDCs (Fig. 7B). Additionally, SFN strongly enhanced the *LPS* up-regulated p50 in the time-dependent manner of *LPS* stimulation apart from at 12 h (Fig. 7B). Besides, TBP was remarkably up-regulated following 24 h of SFN incubation in moDC (Fig. 7C). Likewise NF-κB1 mRNA expression, SFN significantly impaired *LPS*-induced TBP expression at 1 h, but SFN enhanced *LPS*-induced TBP up-regulation at 6 and 12 h (Fig. 7C).

### SFN activates the NF-κB signaling and supresses the cytokines secretion in response to LPS treatment, while enhancing the cellular cytokine accumulation in moDCs lysates

**Fig 8:**
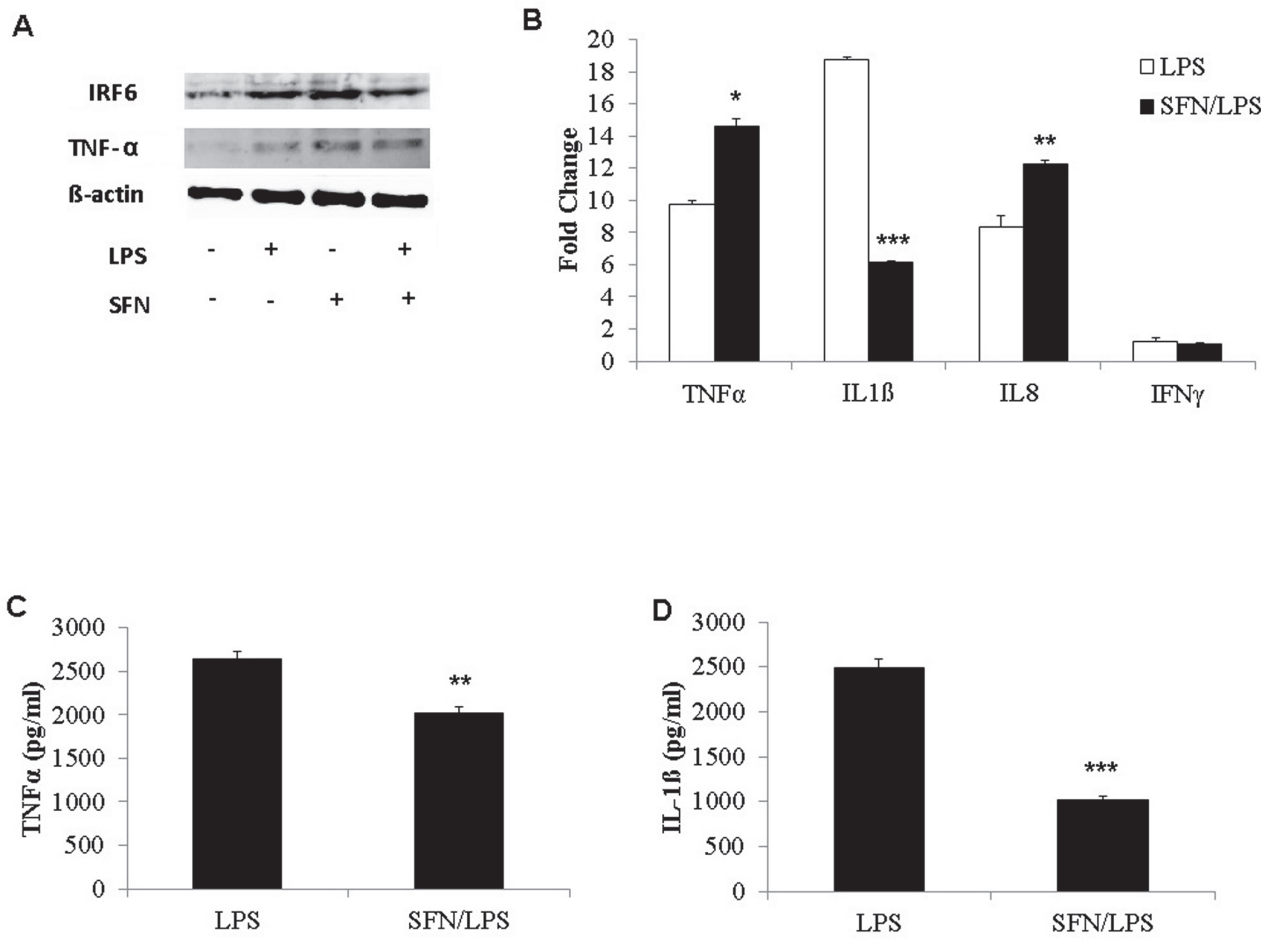
SFN affects gene expressions and protein productions of cytokine. moDCs were pre-incubated for 24 h with or without SFN (10 μM) before stimulation with LPS (1 μg/ml) for additional 24 h. The cell lysate proteins of IRF6 and TNF-α were analyzed using western blotting (A). The effects of SNF on gene expression of pro-inflammatory cytokines TNF-α, IL-1ß, IL-8, and IFN-γ were quantified by qRT-PCR (B). The pro-inflammatory cytokines TNF-α (C) and IL-1ß (D) secreted in cell culture supernatant were determined by ELISA. The results were combined from three independent experiments and each experiment was performed in triplicates. The data were represented as the mean ± standard deviations (SD) (* *P* < 0.05; ** *P* < 0.01; *** *P* < 0.001).

The NF-κB transcription factor has a crucial role in the rapid response to pathogens through modification of down-stream immune gene expressions. The effects of SFN on NF-κB and down-stream protein of immune genes (such as IRF6 and TNF-α) secretion in response to *LPS* treatment were determined using western blotting. The western blotting results displayed that IRF6 secretion was significantly increased in response to either SFN and/or *LPS* compared to control group (Fig. 8A). Moreover, the SFN pre-treatment suppressed IRF6 production in response to *LPS* stimulation (Fig. 8A). According to the western blotting data, TNF-α was remarkably increased in response to either SFN or *LPS* (Fig. 8A). Similarly, SFN pre-treatment further increased the TNF-α production when compared to the moDCs that were not pre-treated with SFN (Fig. 8A). Additionally, several down-stream mRNA expressions of cytokines were quantified using qRT-PCR. SFN significantly up-regulated TNF-α and IL-8 mRNA expression in response to *LPS* treatment (Fig. 8B). On the other hand, SFN remarkably down-regulate IL-1ß mRNA expression in response to *LPS* stimulation (Fig. 8B). SFN had no significant effects on IFN-γ expression in moDCs stimulated with *LPS* (Fig. 8B). Furthermore, TNF-α and IL-1ß protein secretions in moDCs culture supernatant were measured using ELISA. *LPS* significantly increased cellular TNF-α protein production (Fig. 8A), while pre-treatment with SFN significantly decreased secretory protein expressions in supernatant (Fig. 8C and 8D). Notably, TNF-α production in cell culture supernatants measured using ELISA, and mRNA expression in cell lysates quantified using qRT-PCR did not coincide (Fig. 8B and 8C). IL-1ß mRNA expression and protein production was significantly decreased in SFN pre-treated moDCs in response to *LPS* (Fig. 8B and 8D).

## Discussion

DCs are the master phagocytes and antigen-presenting cells (APCs) that bridge the innate and adaptive immunity [34]. Because of the unique ability, DCs can identify pathogens directly by expression of a collection of pattern recognition receptors (PRRs) on the cell surface including TLRs [35]. Although our understanding of the host-pathogen interactions at molecular level has expanded dramatically in recent years, it is believed that the interaction of DCs with pathogens leads to changes in inducible gene expression. The precise molecular and cellular mechanisms of innate immunoregulation through epigenetic changes which responses to pathogens are not yet well understood. SFN represents both anti-inflammatory function and HDAC inhibitory activity [36]. Indeed, histone acetylation is critical for regulation of gene expression in different immune processes. moDCs could be an ideal cell model to unravel the immunoregulatory and HDAC inhibitory effects of SFN in response to *LPS*. This study assayed the ability of SFN to influence the expression of DNMT and HDAC family genes, as well as the effects of SFN on the differentiation and functional properties of moDCs under *LPS*-induced inflammatory model through the TLR4-dependent signalling pathway.

Initially SFN is best known for its role as an indirect antioxidant to process anti-inflammatory activity [37]. Exposure to different SFN concentrations and exposure periods reported in a transient reactive oxygen species (ROS) burst and caused cell death [38,39]. Higher dose of SFN (above 10 μM) was reported to increase ROS levels, which correlated with apoptotic endpoints and cell viability decline [39]. Indeed, our finding is consistent with the above study that the high dose of SFN (such as above 15 μM) significantly induced moDCs cell death. Another potential mechanism of SFN action via SFN-conjugates is histone deacetylase inhibition, which leads to increase histone acetylation. Results of this present study is coincided with our previous research using similar concentration, demonstrated an inhibition of HDAC activity at 10 μM [23]. An aim of the present work is to find an advisable SFN concentration to protect the cell death and play the HDAC inhibitory action to increase histone acetylation. Based on the present data and previous results, 10 μM was considered as an ideal concentration to initiate our study. Expectedly, TNF-α, IL-8 and DNMT1 were positively regulated in SFN pre-treated cells in response to *LPS* stimulation. Similar results have been reported earlier that HDAC inhibitor Trichostatin A enhanced *LPS* induced COX-2, CXCLl2 and IFIT2 expression in macrophages [40]. Besides, IL-1ß was found to be negatively regulated in this study. The up-regulation of TNF-α and IL-8 mRNAs by *LPS* stimulation might be related to the down-regulation of HDAC1 and HDAC2 through NF-κB signal, whereas the subsequent down-regulation of IL-1ß mRNAs in *LPS* induced moDCs might be related to the up-regulation of HDAC9 and HDAC10 mRNAs [41]. Furthermore, SFN inhibited *LPS* induced TLR4/MD2 gene complex and the relevant transcription factors expression at the early stage of stimulus (within 6 h). Along with the previous reports, it could be postulated that SFN pre-treatment down-regulated the TLR4 signalling through the impairing of oligomerization process in a dose dependent manner, leading to the suppression of NF-κB activation [13,15]. But, interestingly and unexpectedly, SFN dramatically enhanced the *LPS* induced relative immune genes expression under longer pathogen stimulation (such as TLR4 and MD2), which is poorly understood.

Epigenetic mechanisms have been shown to play essential roles in the maintenance of gene expression patterns during embryogenesis and cancer [42], but little is known about the roles in immune response in pigs. The steady state levels of acetylation of core histones result from the balance between the opposing activities of histone acetyltransferases and HDACs [43]. HDAC alteration in relation to the aberrant gene expression observed in immune response becomes a critical component in epigenetic mechanism to understand the immune system. In fact, both class I and class II HDACs are involved in regulating proinflammatory response as well as cell proliferation and cell differentiation. HDAC1 and HDAC2 proteins of the class I HDAC are associated in part with the regulation of the transactivation function of NF-κB. Moreover, the association of NF-κB with the HDAC1 and HDAC2 proteins may supress the expression of NF-κB-regulated genes [41]. The finding of this study coincided with the previous report suggesting that that *LPS-*induced down-regulation of HDAC1 and HDAC2 might contribute to the activated NF-κB dependent inflammatory gene expression levels [41]. The class II HDAC has been identified as a general mechanism to control the cytokine production [44]. Previous studies have demonstrated that HDAC6 plays an essential role in regulation of inflammatory immune response in APC/T cell [45], macrophages response [46] and in the cae of atypical airway inflammation [47]. Moreover, a recent study has elaborated that HDAC6 inhibition represents a novel molecular target to disrupt the anti-inflammatory STAT3/IL-10 axis in the APC [48]. Along with the previous results, the downregulation of *LPS*-induced HDAC6 by SFN may postulate anti-inflammatory and anti-tolerance immune response in DCs. Comparatively, the HDAC9 and HDAC10 have less established roles than HDAC6 in immune system, although the inhibition of HDAC10 may regulate HSP-90 acetylation [48]. Besides, HDAC9 and HDAC10 reflect a homologous recombination [49]. Although it is not yet clear whether this is by direct participation or transcriptional control, in accordance with other studies, our data suggest that the effect of SFN on *LPS*-induced upregulation of HDAC10 return to normal phenomenon might be due to the anti-inflammatory function of SFN. Notably, DNA methylation is another key component of epigenetic mechanism that regulates transcriptome levels. In the case of DNA methylation, DNA methyltransferases (DNMTs) are either involved in establishing methylation (i.e., the “*de novo*” methyltransferases DNMT3a and DNMT3b) or copying methylation patterns to the newly synthesized DNA strand during replication (i.e., the “maintenance” methyltransferase DNMT1) [50]. DNMT1 is considered to be the key maintenance methyltransferase in mammals [50]. In this study, DNMT1 was unaffected by *LPS* treatment, but it was increased in SFN pre-treated moDCs in response to *LPS* treatment. These findings indicate that SFN as a HDAC inhibitor might contribute to the suppression of pro-inflammatory cytokines production. In contrast, DNMT3a mRNA was significantly down-regulated by *LPS* stimulation, and further down-regulated in SFN pre-treated moDCs in response to *LPS*. DNMT3a encoding the *de novo* methyltransferases mediates methylation-independent gene repression [51]. The findings of this study coincided with the previous study reported that DNMT3a deficiency leaded to increase cytokine gene expression and resulted in higher inflammatory response in a murine model [52]. We speculate that the regulations of DNMTs methylation might play a role in the immune response to *LPS* with or without SFN pre-exposure. The expression of inflammatory cytokines and other immune genes has been reported to be dependent on methylation status changes at their promoters in human and mouse [52,53]. The combined inhibition in the expression of these deacetylases and DNA methyltransferases could facilitate the transcription of genes in response to *LPS* treatment [54], which suggested that epigenetic factors might be one of the components involved in the regulation of inflammatory response in porcine immune system.

The HDAC inhibitor SFN supressed the *LPS* induced of HDAC gene expressions in this study. Additionally, SFN altered the DNMT1 and DNMT3a expression in porcine moDCs. These data indicate that histone deacetylases positively influence the expression of relevant protein-coding genes. In case of HDAC activity, the addition of SFN to moDC cultures globally inhibited the HDAC activity in a dose-dependent manner. This inhibition of HDAC activity by SFN is coinciding with a previous study [19]. We speculated that alteration in immune gene expression might be related to the SFN induced inhibition of HDAC activity.

DCs play an essential role in the phagocytosis and antigen-presenting that bridges the innate and adaptive immune response. DCs are currently divided into tolerogenic immature and immunogenic mature stages. After stimulation, the immature DCs transform into immunogenic mature DCs, representing unique inducers ready for primary T-cell responses [55]. The foreign antigens can be phagocytized by immature DCs through the interaction of pathogens and the surface receptors on DCs. Immature DC shows the low expression of co-stimulatory molecules CD40, CD80, and CD86. In this study, SFN increased the phagocytic activity in response to *LPS* stimulation in a dose-dependent and inhibited the expression of mature cell surface markers CD80 and CD86 indicating that SFN inhibited the moDCs maturation. This finding coincided with a previous study reporting that HDAC inhibitor led to a tolerogenic phenotype of DCs in mice [56]. This might indicate that SFN enhanced the maintenance of immature moDCs. In a previous study [24], we have shown that phagocytosis of moDCs induced the apoptotic cell death when stimulated with *LPS*. *LPS* binds to TLR4 in complex with MD2, and this complex recruits TGF-ß-activated kinase 1 (TAK1), leading to the activation of NF-κB and consequent transcription of a range of genes coding for proinflammatory cytokines, including TNF-α, pro-IL-1ß and IL-8 [57,58]. SFN-dose is reported to interrupt the engagement of *LPS* in TLR4/MD2 complex and a beneficial anti-inflammatory effects of SFN on TLR4 signalling has been reported previously [13,15]. Therefore, we conducted experiments to see whether the SFN influenced *LPS*-induced TLR4/MD2 complex signalling genes expression on moDCs. For this purpose, we have analysed the expression of TLR4/MD2 complex genes and transcription factor genes in *LPS*-activated moDCs in time-dependent manner after SFN treatment. Additionally, we have determined the protein levels of transcription factor in different time points. Although it is not completely understood how SFN dynamically regulates the immune gene expression, the inhibition of immune genes in the early hours of *LPS* stimulation seems to contribute for the anti-inflammatory function in TLR4 signalling.

Apoptotic function of DCs play a critical role in maintaining a balance between tolerance and immune reaction. The immature DCs start apoptosis and subsequently turn into tolerogenic DCs along with TGF-ß1 secretion and Fox3^+^ regulatory T cells induction [59]. TGF-ß1 is a multipotent cytokine that regulates several pathophysiological events and the secretion of TGF-β1 points out the pathophysiological status of DCs [60]. In this study, the inhibition of *LPS*-induced TGF-ß1 production by SFN might demonstrate that SFN supressed the *LPS* induced apoptotic cell death in moDCs through the suppression of TGF-ß1 signalling. A positive correlation between TGF-ß1 signalling pathway activation and induced cell apoptosis has been reported previously [61,62]. Besides, TGF-ß1 has been shown to improve early DCs development *in vitro* and suppression of immature DCs activation and maturation through inhibiting the up-regulation of co-stimulatory molecules CD80 and CD86 leads to the induction of tolerance to subsequent immunogens [60,62]. Indeed, this study found that SFN inhibited *LPS*-induced TGF-ß1 production, DCs maturation, and simultaneously enhanced the phagocytosis activity in porcine moDCs. These results indicate that the SFN treatment partly benefit DCs anti-inflammatory response.

HDAC inhibitors have been reported to interfere the activation of the mitogen-activated protein kinases, IRFs, or NF-κB signal transduction pathways that induced the transcription and production of immune genes [40,63]. On the contrary, HDAC inhibitors (TSA, VPA, and SAHA) have reported to have no effect on ERK1/2 or on NF-κB, IRF3, or IRF7 nuclear translocation induced by *LPS* or Pam3CSK4 [64]. These inconsistent findings may be due to the different species or immunogens. In case of porcine moDCs, we found that IRF6 was increased in response to *LPS* with or without SFN pre-stimulation. Notably, IRF6 protein was supressed in response to *LPS* in the SFN pre-treated moDCs compared to without SFN pre-treated cells. IRF family members, reported to be involved in the induction of genes that encoded type I IFN, could induce cell differentiation and could regulate gene expression in response to pathogens [65]. IRF6 plays functionally diverse roles in the regulation of the immune system. IRF 6 is involved in the immune response process and alters production of serum IFN-γ, IL-10 level and ratio of IFN-γ to IL-10 in pigs [66]. Therefore, we postulated that SFN pre-treatment could influence the *LPS* induced inflammatory cytokine secretion.

The transcription factor NF-κB plays a crucial role in the transcriptional regulation of genes involved in controlling cell proliferation, differentiation, apoptosis, inflammation and stress responses [67]. Transcriptional modification mediated by HDAC inhibitor SFN may also rely on the acetylation of NF-κB or on the molecules involved in NF-κB signal transduction pathway to control the extent, potency, and duration of NF-κB-mediated transcriptional activity [67]. Therefore, we hypothesize that SFN inhibits the expression of NF-κB1 and TBP which acts as a transcription factors for secondary *LPS-*induced cytokines, such as TNF-α, IL-1ß, and IL-8, in the early stage of inflammation (within 6 h *LPS* stimulation). In most vertebrate cells, NF-κB presents as two (a homo- and heterodimer) structurally related NF-κB proteins, namely p65, and NF-κB1 (p50). In the present study, the cellular NF-κB p65 and p50 protein production was found to increase in moDCs in response to *LPS* stimulation. Notably, NF-κB p65 protein secretion was increased in SFN pre-treated *LPS* induced moDCs compared to only *LPS* stimulated cells. Consistent with the role of NF-κB, this implies that pre-treatment with SFN might contribute to the *LPS* induced inflammatory response. Indeed, SFN pre-treatment suppressed the pro-inflammatory cytokine TNF-α and IL-1ß secretion into the cell culture supernatants, while the cellular TNF-α protein and TNF-α mRNA were increased in this study. It is well known that TNF-α is a proinflammatory cytokine that is rapidly produced following infections, resulting in the initiation of a pro-inflammatory cytokine cascade which can have both beneficial and detrimental effects [68]. The absence of TNF-α bioactivity correlates with an inability to clear the infectious agent resulting in the significant increase of mortality [69,70]. In contrary, excessive TNF-α production in systemic bacterial infection or sepsis is also resulting in an increased mortality [71–73]. Therefore, the present results indicated that suppression of *LPS* induced proinflammatory cytokines expression by SFN might have a beneficial effect on bacterial infection.

In conclusion, to the authors’ knowledge, the present study firstly identify that the HDAC inhibitor SFN regulates the expression of immune genes critical in porcine moDCs responding to bacterial pathogens. Inhibition of HDACs affects the differentiation from immature to mature moDCs, reduces excessive proinflammatory cytokines expression and increases cellular-resident TNF-α accumulation that might enhance pathogen engulfment and clearance through the activation of NF-κB signalling pathway. Additionally, *LPS* stimulation was found to alter the expression of genes encoding epigenetic enzymes in porcine moDCs; however, further studies are needed to confirm exactly how SFN affects various HDAC family members and their individual protein targets. Although, the role of epigenetics in the orchestration of the immune response in porcine immune cells is poorly understood, regulation of the inflammatory response by histone modifying enzymes may focus on the host-pathogen interactions and disease susceptibility.
